# Severity of age-related macular degeneration at first presentation in Bhutan: a 3-year national study

**DOI:** 10.1186/s12886-022-02520-w

**Published:** 2022-07-09

**Authors:** Bhim B. Rai, Michael G. Morley, Paul S. Bernstein, Ted Maddess

**Affiliations:** 1 John Curtin School of Medical Research, Australian National University, 131 Garran Road Acton, Canberra, ACT 2601 Australia; 2 Former Retinal Surgeon, JDW National Referral Hospital, Ministry of Health, Royal Government of Bhutan, Thimphu, Bhutan; 3 Ophthalmic Consultants of Boston, Harvard Medical School, Boston, MA USA; 4 Moran Eye Center, University of Utah, Salt Lake City, Utah USA

**Keywords:** AMD presentation, AMD profile, AMD registry, AMD screening program, Severity of AMD

## Abstract

**Background:**

Medical services are still developing in Bhutan. There is no published national report on age-related macular degeneration (AMD). We therefore aim to determine the demographic characteristics and severity of AMD at first presentation among Bhutanese patients attending their recently inaugurated vitreoretinal (VR) clinics over a 3-year national survey, and to inform national health policy to develop suitable health program to prevent AMD-related blindness and visual impairment.

**Methods:**

A retrospective cross-sectional consecutive case series study was conducted on all new AMD cases in Bhutan. If a patient presented with asymmetrical AMD, the eye with more severe AMD was considered. If both the eyes had the same severity one eye was chosen randomly. Collection of demographic data and clinical details including diagnostic testing (fundus photography, OCT and fluorescent angiography) and clinical staging were performed.

**Results:**

Of 521 new AMD patients aged 71.9 ± 11.3 years, 306/521 (58.7%) were males (*p* = 0.005). At their first presentation, 234/521 patients (44.9%) already had late-stage AMD. Importantly, 69/234 patients (29.5%), that is half of total neovascular AMD (nAMD) patients, had disciform scars (DS) which were beyond treatment, and 7/234 patients (3.0%) had geographic atrophy (GA). Seven patients had retinal pigment epithelium tear at presentation. Fourteen of nineteen polypoidal choroidal vasculopathy (PCV) patients were younger than 50 years.

**Conclusions:**

Half of nAMD cases presented as DS not amenable to the treatment. Many potentially treatable nAMD patients had already lost central vision and were legally blind. Young people with PCV losing vision early in life with longer morbidity-affected life and socio-economic burden was concerning. GA and DS cases need visual rehabilitation to improve their QoL. Incorporating a screening program for AMD with effective health education, and maintaining a national AMD Registry, would potentially lower AMD-related blindness and visual impairment.

## Background

Age-related macular degeneration (AMD) is the leading cause of irreversible blindness in people aged 50 years or older in the developed world [1, 2]. It is caused by both genetic and environmental factors [3]. Age greater than 50 years and a family history are a non-modifiable risk factors, while smoking is a modifiable risk factor [4, 5]. The burden of AMD measured by a loss of quality of life (QoL) is significant: 17 to 60% decrease in the average patient [6]. The total cost of vision loss from AMD was estimated to be $5,128 million in Australia in 2010 [7], $391 million in New Zealand in 2016 [8], and approximately a $30 billion annual negative impact in the USA [6].

In low-to-middle income (LMI) countries, the first presentation of AMD cases depends on many factors: low literacy rate and understanding level of patients, their knowledge and understanding of eye health and diseases, involvement of dominant or non-dominant eye [9], belief in myths and traditional healing, and availability and accessibility to standard eye care services. Bhutan is one such country. Our previous publication reported that 51.9% of patients attending a vitreoretinal subspecialty clinics (VRSC) did not attend modern schooling [10], and the literacy rate among population aged 15 years and above was only 66.6% in 2017 [11]. AMD was the fourth most common retinal disorder in Bhutan and 43.6% of those patients were late-stage at their first presentation [10]. Similarly, AMD was the most common retinal disorder presenting to a tertiary eye care centre in Nepal [9].

Considering the negative impacts of AMD, especially in its late-stages, we decided to investigate AMD severity at the first presentation to the Bhutanese National Vitreoretinal (VR) Services and inform the national health policy with this first study of its kind in Bhutan.

## Methods

### Study design

This was a retrospective cross-sectional case series study approved by the Research Ethics Board of Health (REBH), Ministry of Health, Royal Government of Bhutan, Thimphu, Bhutan which adhered to the principles of the Declaration of Helsinki. Informed consent was waived by the REBH because this retrospective study collected only de-identified data. The source of data was the clinical register maintained by the VR unit. The patient files, OCT scans, FFA films, fundus photographs and neuro-imaging reports were reviewed whenever it was necessary to complete or cross-check the accuracy of data. It is important to note that three of the authors (BBR, MGM, and PSB) were the treating physicians at the time.

### Setting

The study included all new AMD cases presenting to the VR Subspecialty Clinic (VRSC) in Bhutan. The main site was the VRSC at the Jigme Dorji Wangchuck National Referral Hospital (JDWNRH), the apex national referral and a teaching hospital with multispecialty services located in Thimphu, the capital city of Bhutan. The other sites included other VR clinics in Bhutan such as the eastern (ERRH) and central regional referral hospitals (CRRH). The VR services in the country are provided regularly only at the JDWNRH, and periodically in the ERRH and CRRH during visits by the retinal specialist from the JDWNRH. All the VR patients across the country are referred to JDWNRH for management which may involve arduous, long-distance travel.

### Participants

All new AMD patients presenting to the VRSC in whole of Bhutan for over three years (01 May 2013 until 30 April 2016) were included in this study. If a patient presented with asymmetrical AMD, the eye with more severe AMD was used, and if both eyes were symmetrically affected, one eye was chosen randomly. The randomisation was done by tossing a coin, head for right eye and tail for left eye.

We have included 19 cases of polypoidal choroidal vasculopathy (PCV) because it is recognised as a phenotype of AMD [12–14], and both conditions are treated with intravitreal injection of anti-vascular endothelial growth factor (anti-VEGF) [15].

### Clinical examination and data collection

The collected data included demographic information, presenting complaints and their duration, treatments received before presenting to the VRSC, and associated systemic diseases. Best corrected visual acuity (BCVA) and intraocular pressure (IOP) were measured. The anterior and posterior segments were evaluated with slit-lamp and 90D biomicroscopy. The funduscopic findings were evaluated by binocular indirect ophthalmoscopy. Macular and retinal nerve fibre layer (RNFL) scans were done using a Spectral Domain optical coherence tomography (OCT) (Cirrus-HD 4000, Carl Zeiss, 73,447 Oberkochen, Germany). Fundus photographs were taken by a VISUCAM-524 (Carl Zeiss, 73,447 Oberkochen, Germany). AMD was classified by the Clinical Classification of Age-related Macular Degeneration method [16]. The details of the diagnostic tests performed for the study are provided elsewhere [10]. In brief, OCT was performed on all the AMD patients. Fundus macular photography was taken on 331 patients and fundus fluorescein angiography (FFA) performed on 23 patients.

OCT scans, fundus photographs and FFA films were assessed by three retinal specialists (authors BBR, MGM, and PSB) independently, while CT-scans and MRI findings were assessed in consultation with the radiologists, treating neurosurgeons, and other medical specialists.

### Analysis

The data were analysed using MATLAB (2016b, The MathWorks, Natick, MA, USA). Comparisons of the expected and observed frequencies used Chi-squared tests. Logistic regression (LR) used a generalised linear model (Matlab, fitglm) to summarise independent determinants of AMD, late AMD, or end-stage disease such as disciform scar (DS) and geographic atrophy (GA). We formed logical vectors defining these two disease severities and performed LR on them. We have categorised the patients into rural and urban settings. In Bhutan, urban areas were those regions that were under the jurisdiction of City Corporations, municipalities, or town committees, locally termed as *Thromde*, collectively. Other regions were classified as rural areas [17].

## Results

### Demography

A total of 521 new AMD patients were included. Age at presentation was 71.9 ± 11.3 years, and the age range was 20–91 years. The majority of the patients were males (306, 58.7% cf. 215 females, 41.3%, *p* = 0.005), and 274 (52.6%) were from rural and 247 (47.4%) were from urban settings. Farmers were the most common occupation (239, 45.9%), followed by housewives (199, 38.2%), clerical persons (39, 7.4%), retirees (15, 2.9%), government employees (14, 2.7%) and private employees (14, 2.7%). The majority did not attend formal education (435, 83.5%), while only 35 of them (6.7%) attended primary level education, 11 (2.1%) completed high school, and 10 (1.9%) completed postgraduate study. The other demographic details are details are shown in Table 1.

**Table 1:** Demographic characteristics of patients

*Occupation*	*N*	*%*	*Education*	*n*	*%*	*Residence*	*n*	*%*
*Farmers*	239	45.9	Nil	435	83.5	Rural	274	52.6
*Housewives*	199	38.2	Primary	35	6.7	Urban	247	47.4
*Clerical persons*	39	7.4	Graduate	27	5.2			
*Retirees*	15	2.9	High school	11	2.1	**Gender**		
*Govt. employees*	14	2.7	Postgraduate	10	1.9	Males	306	58.7
*Pvt. Employees*	14	2.7	Intermediate	3	0.6	Females	215	41.3
*Student*	1	0.2						
*Total*	521	100		521	100			

Blurry vision was the most common presenting complaint stated by 332 (63.7%), followed by vision not improving post cataract surgery (93, 17.9%), sudden loss of vision (61, 11.7%), and presenting for routine check-up (12, 2.3%).

The severity of the AMD cases is shown in Table 2. At the first presentation, 234 cases (44.9%) were already in the late stages of the disease requiring treatment, or were too late for the treatment. Of them, 69 patients (29.1% of late-stage disease) had a DS, and 7 cases (3.0%) had GA. So a total of 76 cases (32.1%), had DS or GA, and were at a stage for which no currently available treatment could restore functional vision. Nineteen cases (8.1%) were diagnosed with PCV. Fourteen of them were younger than 50 years, including a student aged 20 years. Spontaneous retinal pigment epithelium (RPE) tear was present in 9 patients, giving an incidence of 4.0% p. a. among neovascular AMD (nAMD) including DS, GA excluded.

**Table 2:** AMD severity

*AMD stages*	*n, patients*	*%*
*Early*^a^	269	51.6
*Late*^c^	234	44.9
*Intermediate*^b^	18	3.5
*Total*	521	100
***Late AMD subgroups***	**n, patients**	**%**
*Neovascular AMD*	139	59.4
*Disciform scar*	69	29.5
*PCV*	19	8.1
*Geographic atrophy*	7	3
*Total*	234	100

^a^Early AMD: Medium drusen > 63 μm and ≤ 125 μm and no AMD pigmentary abnormalities; ^b^Intermediate AMD: Large drusen > 125 μm and/or any AMD pigmentary abnormalities;

^c^Late AMD: Neovascular AMD and/or any geographic atrophy [16]

**Fig. 1:**
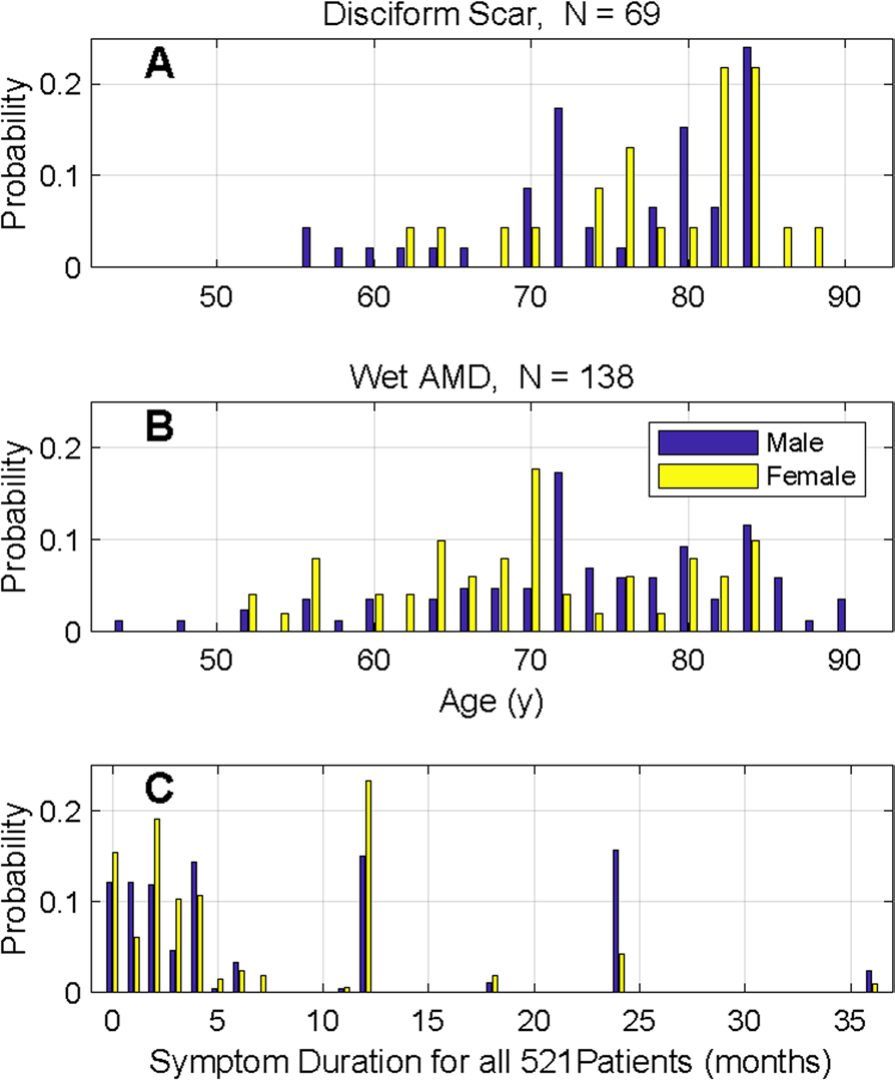
Probability histogram plots by gender. **A** Disciform scar group: a higher proportion of older females are seen in this group than in **B** the Neovascular AMD group. **C** the distribution of symptom duration across all patients in months

As shown in Fig. 1 we split the DS (Fig. 1A) and nAMD (Fig. 1B) eyes into male (blue) and female (yellow). The graphs illustrate that the DS eyes are about half as many as nAMD. They also indicate that there is a higher proportion of older females in the DS group. We verified this with a linear model for age that included three patient groups: DS, nAMD, Others, Gender and an interaction for Group by Gender. That model was significant (F = 6.08, *p* < 0.0001) and showed that while females in the Other group were younger by 3.03 ± 1.25 years (*p* = 0.016) than the males in the Other group, that females in the DS group were older by 6.18 ± 3.08 years (*p* = 0.045). Figure 1C gives the distribution of symptom durations for the whole cohort. The median value was 4 and the interquartile range was 2 to 12 months. The interdecile range was 0.5 to 12 months.

### Logistic regression

The outcomes of LR are the log (Odds), which then are transformed back to the Odds, and their asymmetric 95% confidence limits (columns Lo-CL and Hi-Cl). Table 3 shows the outcomes of the End-Stage disease model (Chi^2^ = 35.9, *p* < 0.0002).

**Table 3:** Logistic regression on End-Stage AMD

	***log(Odds)***	***SE***	***t-Stat***	***p-Value***	***Odds***	***Lo-CL***	***Hi-CL***
*(Intercept)*	0.232	0.565	0.41	0.681			
*Age Relative to Mean*	0.070	0.016	4.27	**0.0001 < **	**1.07 × **	1.04 ×	1.11 ×
*Low Education*	-1.247	0.437	2.85	**0.004**	**0.29 × **	0.12 ×	0.68 ×
*Female*	-0.194	0.296	0.65	0.513	0.82 ×	0.46 ×	1.47 ×
*Urban*	-0.364	0.276	1.32	0.187	0.70 ×	0.40 ×	1.19 ×
*Diabetes Mellitus*	-1.236	0.630	1.96	**0.049**	**0.29 × **	0.08 ×	1.00 ×

The result for age relative to 71.9 ± 11 years was not surprising with risk (odds) of End-Stage AMD rising by 7% per annum (mean of 1.07 × , SE 1.05 × , 1.09 ×), and reducing by that rate below the mean age. Once adjusted for age the other independent effects were less expected: with smaller odds for lower education (Nil or Primary School) of 0.29 × (3.48 × lower), and also smaller odds for diabetes of 0.29 × (3.44 × lower). We made the same model but regressing on Late-AMD for which age and diabetes were not significant. Low education reduced risk was similar to before at 0.40 × (*p* = 0.008). Female gender reduced risk relative to males by 0.66 × (*p* = 0.036). Urban versus rural residence had no effect in either model.

### Associated ocular and systemic co-morbidities

Hypertensive retinopathy was the most commonly associated ocular co-morbidity (33 patients), followed by posterior vitreous degeneration (PVD), non-proliferative diabetic retinopathy (NPDR), asteroid hyalosis, retinal vein occlusion, epiretinal membrane, optic atrophy, glaucoma, macular hole, and solar retinopathy. Associated systemic disorders included hypertension (123 patients), diabetes (49 patients), co-existent hypertension and diabetes (41 patients), congestive cardiac failure (2 patients) and chronic renal failure (2 patients).

### Prior interventions

These interventions were performed in other countries before the patients presented to the VRSC in Bhutan. Among the study patients, cataract surgery was the most common intervention performed in 74 patients. Intravitreal injection of anti-VEGF was performed in only 6 cases, 4 patients received injection ranibizumab (Lucentis) and 2 patients received injection bevacizumab (Avastin). Two patients had received retinal detachment surgery, and one had undergone trabeculectomy.

### Treatment of neovascular AMD

In the current study, nAMD, including PCV, was treated with anti-VEGF at the JDWNRH. In Bhutan, the medical expense is born by the government. So for economic reasons, intravitreal injection of bevacizumab (Avastin, Genentech, San Francisco, CA, United States) was administered universally, except three cases who received ranibizumab (Lucentis, Genentech, San Francisco, CA, United States). A report on the management of VR diseases including AMD in Bhutan is published elsewhere [18].

## Discussion

In LMI countries particularly, late presentation for health consultations and late diagnosis not only compromises the effectiveness and outcomes of treatment, but they also increase the costs and socio-economic burden due to blindness and visual impairment and impair QoL including mental health [19]. The most common issue is poor availability and accessibility of health services in a region, as observed in our study. Bhutan did not have VR services until early 2012, when the first national retinal specialist (author BBR) joined the national service and established the VR Unit and National Vitreoretinal Services including hospital-based clinical, surgical and laser services [10, 18, 20]. Before that period, VR cases were referred outside of Bhutan, mostly to India [10]. Many patients were reluctant to go abroad for treatment, however, resulting in frequent loss to follow up, with some seeking traditional healing practices [21]. This might be the reason why only 4 AMD patients in this study had received anti-VEGF injection as prior interventions despite a high prevalence of late-stage AMD cases in the country. Injected anti-VEGF is the mainstay of nAMD treatment, yet such treatment was rarely received by the study patients. However, after the service was established, administration of anti-VEGF injections for nAMD became the most frequent intervention performed in the VR operating theatres, as mandated in Bhutan and other LMI countries like India and Nepal in the region [18].

In LMI countries, illiteracy and ignorance might have been other barriers for early presentation [19]. Our study reveals that 83.5% of the study patients had not received modern schooling and the majority of them were farmers and housewives. Similarly, our previous study also reported that 51.9% of the people reporting for VR services did not attend modern schooling [10]. Compounded by lack of health facilities and accessibility, this group of people could be easily influenced by local healers and might have had less faith in modern medicine [22]. That being said, lower education appeared to be protective (Table 3), although that might be due to some confounder like diet. Yet another barrier could be the easy availability of alternative treatment approaches. In Bhutan, most Bhutanese are happy to receive treatment from, or have good faith in, the traditional medical system known as *sowa rigpa*, meaning “the science of healing”. *Sowa rigpa* is popular among Bhutanese and most of them are satisfied with it [21]. Although most people are satisfied with that treatment it may be a reason for late presentation, poor visual recovery, and ultimate loss of vision from AMD.

The most concerning fact about AMD patients in Bhutan is that 44.9% of them already had AMD in late stages at their first presentation, and 32.1% of them had either DS or GA. By contrast, in developed countries patients are diagnosed at early or intermediate stages and followed up until late-stage AMD when treatment is started [23]. Figure 1 and associated linear model indicated that DS males were not older than nAMD males or all other males, but females were. This might indicate that females seek treatment later but more data would be required to verify this. However, this is supported by our previous hospital based study in nearby Nepal, which reported that females presented almost a decade later than their male counterparts [9]. The incidence of RPE tear in Bhutan was less than those reported by other studies ranging from 5 to 19.7% [24, 25]. RPE tear is a potentially vision threatening complication of nAMD affecting visual prognosis [26]. Yet another unexpected finding was very low cases of pre-existing GA representing only 3% of advanced AMD. An age-related eye disease study has reported a baseline incidence of 9.8% p.a. and a Kaplan–Meier incidence rate of 19% at 5 years [27]. We have no explanation for this low GA finding. We are hoping to investigate this in a future study.

Yet another point to consider is that the second most common symptom to seek retinal consultation was vision not improving after cataract surgery, second only to blurry vision. Generally, cataract operations are very rewarding with rapid full visual recovery. Vision not improving after cataract operation is a major concern to both the patients and the operating surgeons and is a setback given the patients’ disappointment and dissatisfaction. Such outcomes indicated that most of the patients were not aware that they had AMD, or that it existed. Their diagnosis of AMD was purely incidental. This warrants more public advocacy on what AMD is, its associated factors, treatment availability, and the benefits of early diagnosis and treatment of AMD.

Age was the most obvious associated factor (Table 3), but with diabetes and low education appearing to be somewhat protective. Loosing these eyes sends out a strong message about the failure of the AMD treatment program considering that in modern times, we claim that we have effective and successful treatments for nAMD [28]. There is no point in having modern well-equipped hospitals in towns and cities if majority of the patients, illiterate and ignorant, reside in regional areas, are not educated about ocular diseases and their treatment options and benefits.

We found that low education and female gender reduced risk of Late-AMD, independent of age. The reason for female gender to have reduced risk for Late-AMD could be that relative to male adults and adolescents, females smoke less in Bhutan [29], and so the males might have increased risk for Late-AMD.

In our study, we found a lower rate of Late-AMD among the patients with diabetes. A review article by Chen et al. [30] reported that East Asians had a slight tendency for protection from AMD by diabetes. Metformin use has been associated with reduced odds of developing AMD, which was also dose dependent [31]. In Bhutan metformin is widely used to manage both pre-diabetic and diabetic patients [32]. Metformin has protective effects against hydrogen peroxide-induced oxidative damage in human RPE cells by enhancing autophagy [33]. It also protects the RPE cells form glyoxal-induced oxidative stress [34]. Atop, this finding might have been due to shorter life expectancy in diabetics, therefore less age to develop AMD [35].

PCV is recognised as a sub-type of nAMD, more common in Asian patients compared to western counterparts [12–14]. If not treated in time it can lead to irreversible vision loss sue to haemorrhage and scarring [36], and decrease vision related QoL [37]. It is estimated that by 2050, PCV will affect more patients in Asia than in the rest of the world [14]. This fact is concerning to Bhutan as fourteen of nineteen PCV patients were under fifty years of age. It is mainly affecting the working-age population, meaning it will impact the economy. Earlier onset means longer morbidity years and more socio-economic burden with longer reduced QoL.

Silent diseases such as AMD and asymptomatic retinal tears are also under-reported in Bhutan [38]. A national survey on retinal laser services in Bhutan revealed only 5.3% of total retinal laser therapy was performed for retinal breaks over three years [20], although myopic patients referred for retinal evaluation for myopic degenerative complications was the second most common reason for seeking retinal consultation [10] and myopia constituted 92.1% of refractive error [39].

Tobacco smoking is a modifiable risk factor [5]. In our study, we found that male gender as an associated factor to more advanced AMD because they smoke more. So in the AMD screening program, health advocacy on the risk of smoking should be highlighted. For effective cessation of smoking, our screening program and health education may be liaised with the anti-smoking campaign in Bhutan [40].

Considering the fact that a large proportion of nAMD patients already had a DS at their first presentation compared to other studies [2, 23], the low understanding of the general population, and difficult terrain to travel through to obtain the VR services, a screening program should be prioritized. To achieve economies we could combine the AMD screening program with diabetic retinopathy screening program and screen all the diabetic patients aged above 55 years for AMD in the beginning and if there is economic barrier. This approach will screen only a subset of potential AMD cases who are diabetics. The optometrists and ophthalmic technicians can be trained to do the AMD screening as they were in the DR screening program, using fundal examination under mydriasis at the community level. In long run, an independent AMD screening program with *AMD Registry* is needed to screen all the eligible population. In any case the data indicate that, given the good availability of anti-VEF therapy in Bhutan, that an AMD screening program would likely reduce the prevalence of DS and nAMD. However, the regular vitreoretinal service centre is located only in the capital city, Thimphu. So this demands developing an effective screening program with convenient treatment program in the regional health centres, or an efficient referral system including travel arrangement. Management by VR specialists can be done in two ways: first, pooled patients or individually can be referred to the JDWNRH, second, if the patients are not willing to travel far, they can be booked and pooled for treatment in the regional referral hospitals on scheduled visits by VR specialists.

One disadvantage in recommendation of the AMD screening program is that the program may create further burden on already limited health resources in a LMI country like Bhutan.

We highly recommend educating Bhutanese population on AMD and the possibility of treatment through mass media such as national television, newspapers and through personnel devices using Facebook.

Our study is limited by being a hospital-based study rather than a population-based study. Therefore, it does not reflect the true prevalence of the various stages of AMD in the community. There may be a regional bias because the JDWNRH, where the VR clinic or VR service is regularly provided is located in the north-west part of the country. Although all AMD patients are ultimately referred to the JDWNRH from all over the country and also some patients were seen in the ERRH and CRRH many patients may still not have been picked up from the eastern and central zones. In the future, we look forward to conducting a population-based study to provide the true prevalence of the AMD in Bhutan. The study also lacks vital information such as family history of AMD and smoking. At this point, we have not evaluated the usefulness of a community screening program for AMD, but we have a plan to conduct such a study in future. That should also include further demographic factors such as lens status, smoking status, and family history of AMD.

## Conclusions

We found that 69 patients, nearly 10/100,000 amongst the small national population of Bhutan [41], were lost to DS due to untreated nAMD. Many of these eyes would otherwise have been very treatable with substantial visual recovery potential if diagnosed earlier. And there are many more people in LMI countries who do not know that they are suffering from silent diseases like AMD. Losing vision or falling sick for them is punishment from God or nature’s cycle. In summary, it may be worth having a community screening program for AMD cases and maintain AMD registry. To achieve economy, AMD screening may be combined with the national diabetic retinopathy screening program in the beginning, which already exists in Bhutan. Later on, a full-fledged AMD screening program is advised. Besides, health advocacy on various eye diseases including AMD should be mandated and reinforced.

## Data Availability

The data have not been placed in any online data storage. The datasets generated and analysed during the study are available upon request from the first author Bhim B Rai.

## Abbreviations

AMD: Age-related macular degeneration
BCVA: Best corrected visual acuity
CRRH: Central regional referral hospital
DS: Disciform scar
ERRH: Eastern regional referral hospital
GA: Geographic atrophy
JDWNRH: Jigme Dorji Wangchuck National Referral Hospital
LMI: Low-to-middle income
nAMD: Neovascular AMD
NPDR: Non-proliferative diabetic retinopathy
OCT: Optical coherence tomography
PCV: Polypoidal choroidal vasculopathy
PVD: Posterior vitreous degeneration
QoL: Quality of life
REBH: Research Ethics Board of Health
RNFL: Retinal nerve fibre layer
VEGF: Vascular endothelial growth factor
VR: Vitreoretinal
VRSC: Vitreoretinal subspecialty clinics
